# Factors associated with unfavorable treatment outcomes among multidrug-resistant tuberculosis patients, Sierra Leone: a cross-sectional secondary data analysis

**DOI:** 10.1186/s12879-024-09370-5

**Published:** 2024-06-11

**Authors:** Josephine Amie Koroma, Adel Hussein Elduma, Umaru Sesay, Gebrekrstos Negash Gebru

**Affiliations:** 1 National Leprosy and Tuberculosis Control Program, Ministry of Health, Freetown, Sierra Leone; 2 Sierra Leone Field Epidemiology Training Program, EOC Building, Wilkinson Road, Freetown, Sierra Leone; 3 African Field Epidemiology Network (AFENET), EOC Building, Wilkinson Road, Freetown, Sierra Leone

**Keywords:** Multidrug-resistant tuberculosis, Unfavorable treatment outcomes, Sierra Leone

## Abstract

**Background:**

Globally, multidrug-resistant tuberculosis (MDR-TB) is a major public health problem. The tuberculosis rate in Sierra Leone is 298 per 100,000 people, and Sierra Leone is considered a country with a high burden of tuberculosis. In Sierra Leone, there are few studies on the outcomes of MDR-TB treatment, especially those exacerbated by COVID-19. We identified factors associated with unfavorable treatment outcomes among people with MDR-TB in Sierra Leone.

**Methods:**

We conducted a cross-sectional study to analyze hospital-based MDR-TB data from 2017 to 2021. Demographic, clinical, and treatment outcome data were extracted from the main MDR-TB referral hospital database. We defined unfavorable outcomes as patients who died, were lost to follow-up, or defaulted. We calculated adjusted odds ratios (aORs) and 95% confidence intervals (CIs) to identify predictors of the outcomes of MDR-TB treatment.

**Results:**

Between 2017 and 2021, 628 people with MDR-TB were reported at Lakka Hospital; 441 (71%) were male, with a median age of 25 years (interquartile ranges: 17–34). Clinically, 21% of the 628 MDR-TB patients were HIV positive, and 413 were underweight (66%). 70% (440) of MDR-TB patients received tuberculosis treatment. The majority of patients, 457 (73%), were treated with a short treatment regimen, and 126 (20%) experienced unfavorable outcomes. Age 45 years or younger (aOR = 5.08; CI:1.87–13.82), 21–45 years (aOR = 2.22; CI:140–3.54), tuberculosis retreatment (aOR = 3.23; CI:1.82–5.73), age group, HIV status (aOR = 2.16; CI:1.33–3.53), and malnourishment status (aOR = 1.79; CI:1.12–2.86) were significantly associated with unfavorable treatment outcomes for DR-TB patients.

**Conclusion:**

This analysis revealed a high proportion of unfavorable treatment outcomes among MDR-TB patients in Sierra Leone. Malnourishment, TB retreatment, HIV coinfection, and age 45 years or younger were associated with unfavorable outcomes of MDR-TB treatment. Increasing patients’ awareness, mainly among young people, heightens treatment adherence and HIV monitoring by measuring the amount of HIV in patient blood, which can reduce adverse treatment outcomes in Sierra Leone and other sub-Saharan African countries.

## Background

Multidrug-resistant tuberculosis (MDR-TB) is a serious form of tuberculosis that develops resistance to isoniazid and rifampicin [1]. This resistance can arise due to inadequate treatment, incorrect use of TB drugs, exposure to people with MDR-TB, poverty, and social risk [2]. Globally, approximately 410,000 individuals were diagnosed with MDR-TB, and 160,000 deaths from MDR-TB were recorded in 2022 [3]. The proportion of MDR-TB cases ranged from 3% in the WHO South‒East Asia, WHO African, and East Mediterranean regions to 54% in the European region [4]. The global burden of MDR-TB is further compounded by the challenges of TB control, including inadequate TB detection and treatment, poor access to healthcare, and limited resources [5]. In 2023, an estimated 77,000 new cases of MDR-TB were reported in Africa [6]. Among those with MDR-TB in Africa, 55% were reported in South Africa, Nigeria.

Between 2000 and 2021, Sierra Leone’s mortality from tuberculosis decreased from 75 per 100,000 in 2000 to 31 per 100,000 in 2021 [7]. This progress was in part due to efforts implemented by the Government of Sierra Leone with the support of its partners. Some of these efforts include the establishment of an MDR-TB treatment center at the National Referential TB Hospital, the capacity building of staff to diagnose MDR-TB, and the adoption of the GeneXpert for TB diagnosis. Since the establishment of MDR-TB treatment in 2017, the country has registered more than 640 MDR/RR-TB patients by the end of 2021 [8]. The treatment coverage is 87% based on the projected population census for 2020 [9]. Despite the progress made in the detection and treatment of tuberculosis, the estimated incidence rate of tuberculosis was 298 per 100,000 people in 2021 [10]. Additionally, the rate of tuberculosis case stratification (CNR) was 213 per 100,000 people. Despite the significant advances made by the country in controlling tuberculosis in recent years, the gaps in drug-susceptible tuberculosis (DST) have led to the development of drug-resistant TB (DR-TB) [11].

Initially, the World Health Organization (WHO) recommended the use of the long MDR-TB regimen, which ranges from 18 to 20 months [12]. Due to the extended duration of drug administration, the Sierra Leone TB program encountered adherence challenges. In October 2016, the country began a standardized short MDR-TB regimen, which is 9 to 11 months long, after which it was approved by the WHO to replace long conventional regimens for people with MDR-TB who have not previously been treated with second-line drugs and for whom resistance to fluoroquinolones has been ruled out [13].

To date, several studies have assessed unfavorable treatment outcomes and associated factors among people with MDR-TB. For example, a study conducted in Ethiopia identified HIV coinfection as the main factor associated with unfavorable outcomes among people with MDR-TB [14]. In Colombia, factors associated with unfavorable outcomes among people with MDR-TB were age older than 60 years and receiving subsidized care [15]. HIV, renal diseases, and diabetes were identified as factors associated with unfavorable treatment outcomes among MDR-TB patients in Papua New Guinea [16]. Another study conducted in the Oromia region of Ethiopia reported that HIV and culture at the end of the continuation phase were factors associated with unfavorable outcomes among MDR-TB patients [17].

Determining the factors associated with unfavorable TB outcomes provides information on the pattern of distribution of unfavorable treatment outcomes in Sierra Leone. Policymakers should be better informed about the design of appropriate interventions and strategies to improve the performance of tuberculosis treatment. This study aimed to determine the prevalence and factors associated with unfavorable treatment outcomes of MDR-TB in Sierra Leone.

## Methods

### Study design

We conducted a cross-sectional study to analyze MDR-TB data collected from Lakka Hospital in Freetown, Sierra Leone, from January 1, 2017, to December 31, 2021.

### Study setting

This study was conducted at Lakka Hospital (a teaching hospital). The hospital is situated in the coastal areas of Freetown, a Western area rural district in Sierra Leone. This hospital is a referral hospital equipped with 149 beds and admits patients with drug-sensitive tuberculosis (DS-TB) and MDR-TB. The hospital has six doctors and 90 nurses, 19 laboratory scientists and technicians, and four radiographers.

### Participants

The study population included people with MDR-TB who completed their treatment regimen and whose data were reported in the MDR-TB database. Patients who were still under treatment and those who were transferred out of the hospital were excluded.

### Data collection

We used MDR-TB data (2017–2021) extracted from MDR-TB registries. We extracted patient demographics (districts and regions, age, gender), clinical symptoms, treatment outcomes, TB/HIV coinfection status, BMI outcome, treatment regimen, and patient category (new and retreated). The treatment outcome was determined by physicians working at Lakka Hospital. The classification was based on the laboratory results, clinical improvement, and adherence to the treatment. All people with MDR-TB during the study period were selected for inclusion in the study. Accordingly, people with MDR-TB were classified as cured, treatment completed, treatment failure, death, or loss to follow-up. We categorized treatment outcomes into binary variables: favorable outcome (cured or treatment complete) and unfavorable outcome (death, loss to follow-up, failure, or not evaluated). Death was defined as any MDR-TB patient who died before starting or during treatment. Failure was defined as MDR-TB patients whose culture was positive at month 5 or later during treatment.

Patients who were lost to follow-up were defined as patients whose treatment was interrupted for 2 consecutive months or more. A lack of evaluation was defined as no treatment outcome. These categories include who was lost to follow-up, who was transferred out, or for whom no treatment outcome information was available. Patients who completed treatment for MDR-TB were defined as those who completed treatment without evidence of failure and for whom records were available. A cured patient was defined as a pulmonary MDR-TB patient with bacteriologically confirmed TB at the beginning of treatment who was smear or culture negative in the last month of treatment and on at least one previous occasion.

Prior knowledge of the data may increase the risk of bias because it can motivate the researcher to pursue certain analyses. To address this bias, we conducted a multiverse analysis to identify all potential analytical methods to address the study research questions.

### Data analysis

We performed descriptive analysis for continuous variables and analysis for categorical variables, including favorable outcomes and unfavorable outcomes. We considered age, sex, HIV status, new and retreatment cases, underweight status, and treatment regimen as the exposure variables. Univariate analysis was used to assess any associations between treatment outcomes and exposure variables. We used 0.2 as the cutoff point for the bivariate analysis to include variables in the multivariate analysis. We used univariate analysis to identify important variables based on the literature to build the logistic regression model. Then, we performed bivariate and multivariate analyses using Epi-info software version 7.2.5 [18]. For bivariate analysis, crude odds ratios (ORs) with 95% confidence intervals (CIs) were calculated. We used 0.2 as a cutoff point to select variables from the bivariate analysis to be included in the multivariate model. To control for confounders, we used multivariate analysis to compute the adjusted odds ratio (aOR) at the 95% CI, and a P value < 0.05 was taken as the cutoff to identify factors associated with the outcome variable. The data were analyzed using Excel and STATA software version 14.

## Results

### Demographic characteristics

**Fig. 1:**
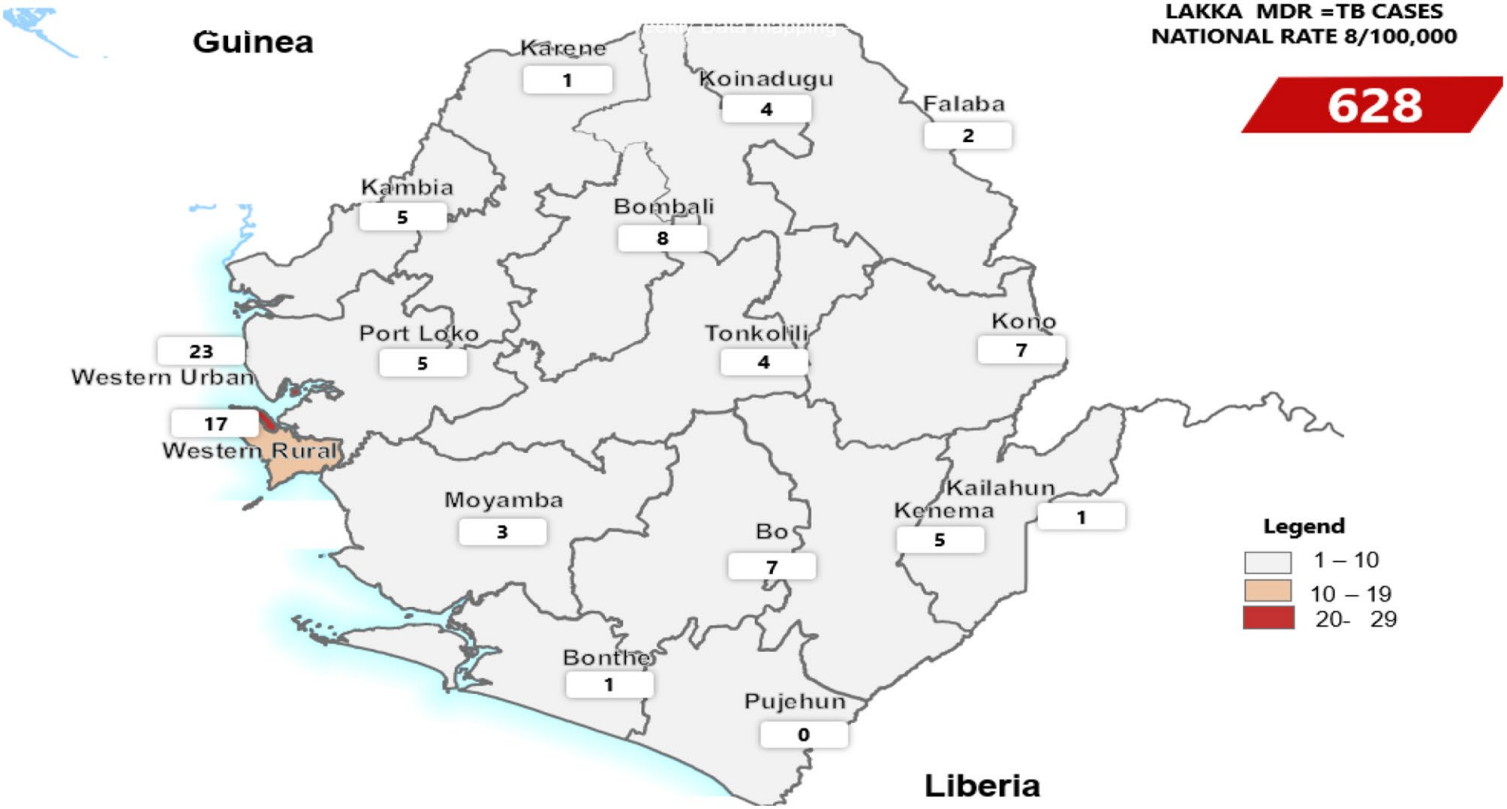
Distribution of MDR-TB cases by district, Sierra Leone, 2017–2021

**Fig. 2:**
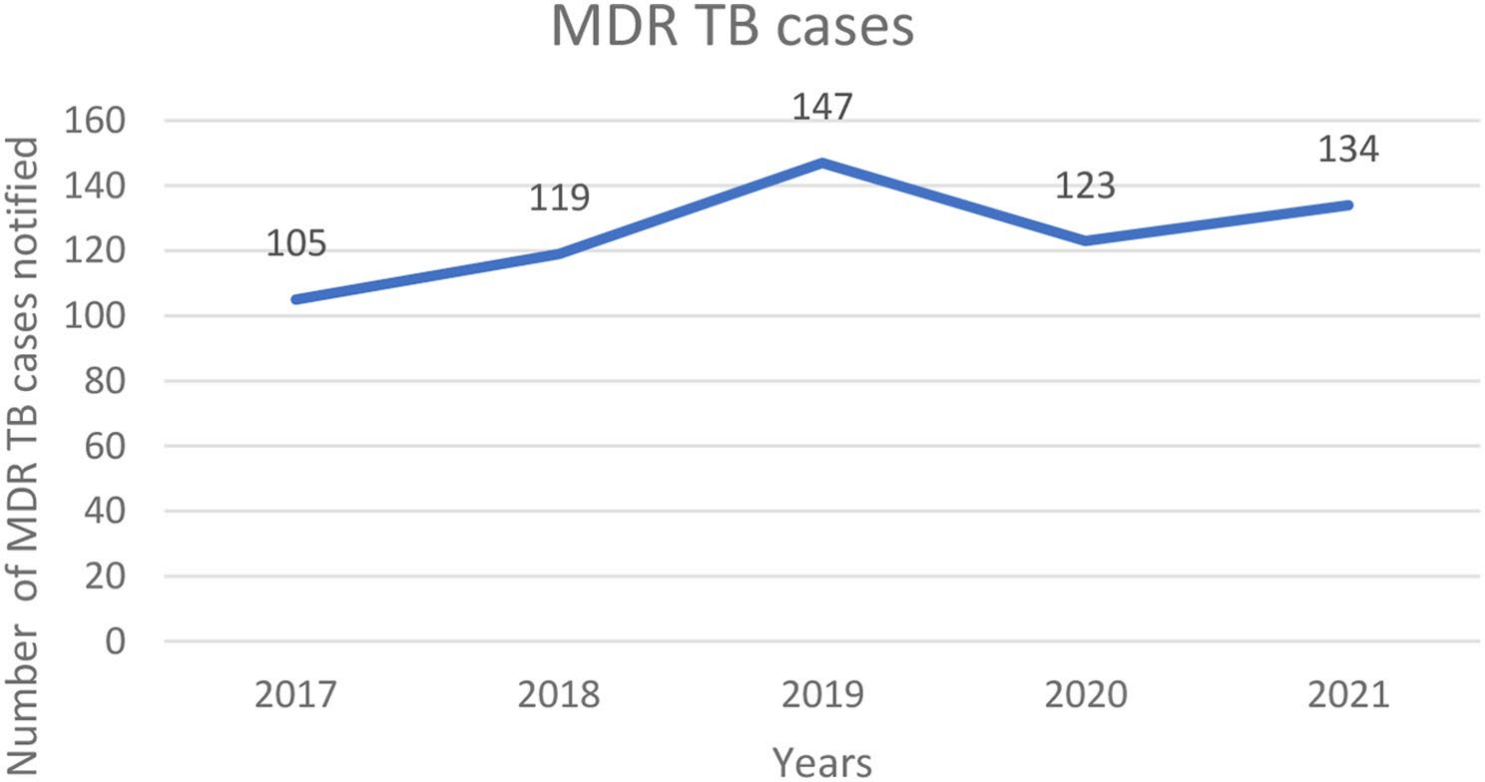
Number of MDR-TB cases by year at Lakka Hospital, Sierra Leone, 2017–2021

**Fig. 3:**
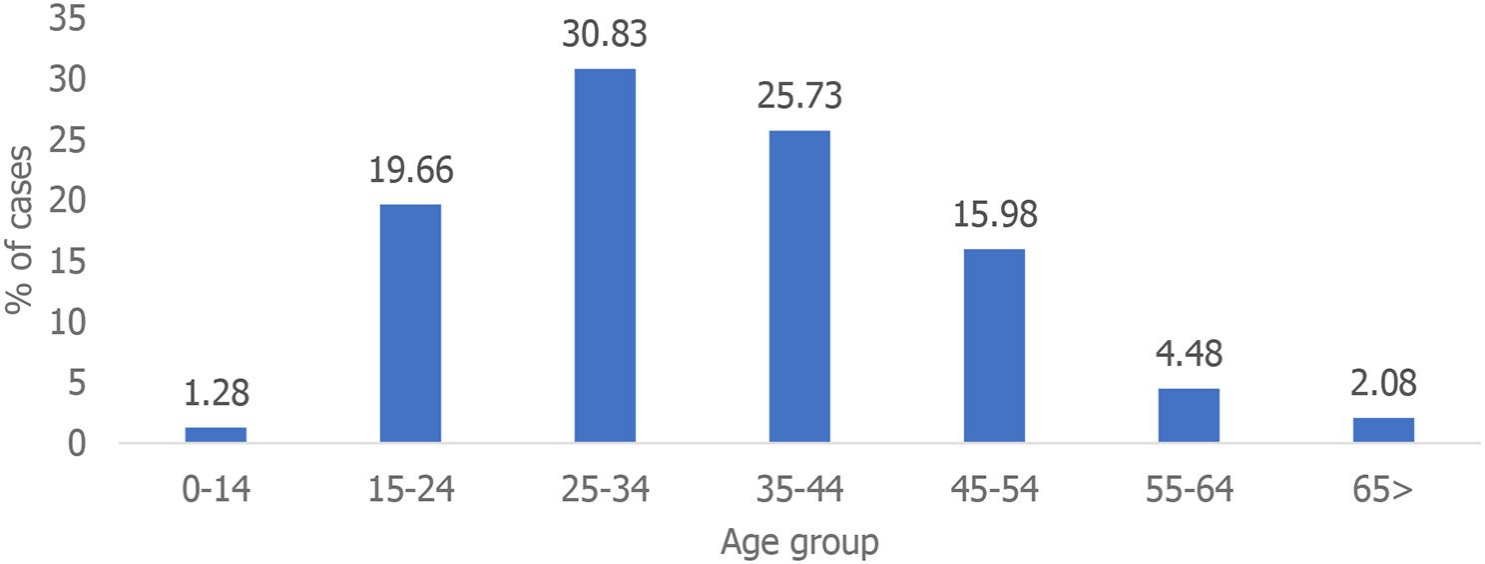
Distribution of MDR-TB cases by age group in Sierra Leone, 2017–2021

A total of 628 MDR-TB patients were recorded during the study period. Males accounted for 70% (440 of 628) of the sample, and the median age was 25 years (interquartile range: 17–34). All sixteen districts reported at least one MDR-TB case (Fig. 1). There were 105 people with MDR-TB in 2017, 119 in 2018, 147 in 2019, 123 in 2020, and 134 in 2021 (Fig. 2). 22% (136 of 628) of the MDR-TB cases were reported in the first quarter (January, February and March), 23% (145 of 628) in the second quarter (April, May and June), 27% (169 of 628) in the third quarter (July, August, and September), and 28% (176 of 628) in the fourth quarter (October, November, and December) of the study period. Most of these patients were aged between 20 and 45 years (61%, 384 of 628), followed by those aged between 1 and 19 years (31%, 197 of 628) (Fig. 3).

### Treatment outcome

51% (322 of 628) of the people with MDR-TB had successful treatment, 66% (411 of 628) were underweight, and 21% (134 of 628) were coinfected with HIV (Table 1). Of the remaining 49%, 21% had unfavorable treatment outcomes (15% died, 3% failed treatment, and 3% were lost to follow-up). The remaining 28% are yet to be determined because their treatment course is ongoing.

**Table 1:** Treatment outcome demographic data of people with MDR-TB in Sierra Leone, 2017–2021

Variables		Years				
		2017	2018	2019	2020	2021
Completed/cured		74.76%	73.98%	75.52%	31.71%	1.49%
Died		16.50%	17.89%	14.69%	21.14%	7.46%
Failed		1.94%	4.88%	3.50%	0.00%	0.00%
LTFU		6.80%	3.25%	2.80%	1.63%	0.00%
On Rx		0.00%	0.00%	3.50%	45.53%	91.04%
TOTAL		100.00%	100.00%	100.00%	100.00%	100.00%
**Gender**		**Frequency**	**Percentage**			
Male		148	29.7			
Female		440	70.3			
**Age group**		**Frequency**	**Percentage**			
1–19		197	31.4			
20–45		384	61.1			
46–65		47	7.5			
**HIV status**						
Negative		494	78.7			
Positive		134	21.3			
**Treatment regimen**						
Long		172	27.4			
Short		456	72.6			

Of the severely underweight patients, 21% (83 of 401) had an unfavorable outcome, while 79% (318 of 401) had a favorable outcome (Table 1). 70% (440 of 628) of people with MDR-TB were retreated, 75% (330 of 440) of whom were males. 85% (374 of 440) of retreated patients with MDR-TB had unfavorable treatment outcomes. Of the people with MDR-TB, 73% (456 of 628) were receiving short-regimen treatment. Of the people with MDR-TB who received TB treatment for a second time, 70% (439 of 628) and 14% (86 of 628) died due to the infection, respectively. 90% (122 of 628) of the people with MDR-TB had unfavorable outcomes; 71% (87 of 122) of them were male. The majority (75%, 94 of 122) of patients with unfavorable outcomes received long-term treatment (Table 1).

### Univariate and multivariate analysis of the factors associated with unfavorable outcomes

According to the univariate analysis, malnourishment, retreatment, HIV coinfection, and age were associated with unfavorable treatment outcomes. Patients in the 20–45 years and 46 years and older age groups were more likely to have unfavorable outcomes (aOR = 1.7; CI = 1.05–2.86 and aOR = 5.2; CI = 2.46–11.17, respectively). Furthermore, tuberculosis retreatment (aOR = 3.2; CI: 1.87–5.69), HIV coinfection (aOR = 2.2; CI: 1.39–3.67), and underweight (aOR = 1.9; CI: 1.23–3.18) were independently associated with unfavorable outcomes (Table 2).

**Table 2:** Bivariate and multivariate analyses of factors associated with unfavorable outcomes in multidrug-resistant tuberculosis patients in Lakka Hospital, Freetown, Sierra Leone

Variable	Unfavorable outcome	favorable outcome	cOR, 95% CI	*P* value	aOR 95% CI	*P* value
**Gender**						
Female	87	353	Ref			
Male	35	151	1.0 (0.68, 1.64)	0.783		
Regimen						
Long	28	140	Ref			
Short	92	364	1.2 (0.74, 1.86)	0.478		
**Age group**						
1–19	26	171	Ref			
20–45	77	307	1.6 (1.01, 2.67)	0.042	1.7 (1.05, 2.86)	0.029
46–65	19	26	4.8 (2.33, 9.88)	0.000	5.2 (2.46,11.17)	0.000
**HIV**						
Negative	86	406	ref			
Positive	36	98	1.7 (1.10, 2.71	0.016	2.2 (1.39, 3.67)	0.001
**BMI outcome**						
Normal weight	29	186	Ref			
Underweight	83	318	1.8 (1.19, 2.95)	0.007	1.9 (1.23, 3.18)	0.005
**Treatment status**						
New treatment	18	169	Ref			
Retreatment	104	335	2.9 (1.70, 4.96)	0.000	3.2 (1.87, 5.69)	0.000

## Discussion

In this paper, we determined the magnitude and factors that contribute to unfavorable treatment outcomes among MDR-TB patients in Lakka Hospital, Sierra Leone. The average treatment success rate found in our study (51%) was below the global target of < 75% [19]. This was consistent with a study conducted in Uganda and Ethiopia, where the authors reported treatment success rates of 71.8% and 64.7%, respectively, which were also below the global target [20, 21]. Although the average treatment success rate reported in our study was below the global target, our findings revealed that there was a gradual increase in the treatment success rate before the emergence of COVID-19. A study conducted by Alena and colleagues [22] reported that the global pandemic of COVID-19 may have ramifications for the global effort to eradicate tuberculosis by 2035. The authors further reported that shortages of resources, either directly from pandemic management or indirectly from the pandemic’s larger economic repercussions and stretched national budgets, are likely to influence ordinary public health programs, including tuberculosis prevention and control activities.

Our study revealed a high prevalence of MDR-TB in people at Lakka Hospital, Sierra Leone, and the majority of the cases were reported in the Western Area Urban District. The high burden of MDR-TB in the Western Area Urban District relative to the other districts could be attributed to several factors. The Western Area Urban District hosts the main capital city of Sierra Leone, Freetown, and hosts the majority of the Sierra Leone population, with an estimated 15% (1,055,964 of 7,092,113) according to the 2015 national census [23]. Poor environmental sanitation and congested housing could be the factors that contribute to tuberculosis transmission [24]. , our study suggested that this could be the main reason for the high burden of people with MDR-TB in Sierra Leone and the Western Area Urban District in particular. This finding was consistent with a study conducted in Tanzania, where the majority of MDR-TB patients resided in Dar es Salaam, the capital [25]. This might be attributed to an increased risk of contracting MDR-TB infection due to overcrowding.

Additionally, patients aged 45 years and older were more likely to experience unfavorable treatment outcomes than patients in the other age groups were. As revealed in several other studies [26–28], patients aged 46 years and older are prone to underlying medical conditions such as hypertension and diabetes, malnutrition, and immunosuppressive therapies, among others. It is worth mentioning that the cost of managing tuberculosis patients with comorbidities in Sierra Leone is high, making it difficult to achieve successful treatment outcomes for people suffering from these conditions [29].

Additionally, patients with HIV, malnourishment, and retreatment were more likely to have unfavorable treatment outcomes than their counterparts. These factors have been reported in several studies to compromise the immunity of patients suffering from tuberculosis, hence making them susceptible to unfavorable treatment outcomes. Another study on the social determinants and comorbidity of tuberculosis reported that HIV-infected patients have a greater risk of contracting tuberculosis than those without HIV [30]. The study also reported that the risk is even greater among patients with a lower CD4 + T-cell count and suggested a multisectoral approach to detect people suffering from the condition to lower the risk of adverse treatment outcomes. In contrast, a study conducted by Larkoh and colleagues reported that patients with negative HIV status had 2 times greater odds of having unfavorable treatment outcomes than those with positive HIV status [22]. Our findings showed that previous treatment with TB drugs is associated with unfavorable outcomes among people with MDR-TB. A similar finding was reported in a study conducted in Vietnam, where previous treatment courses and HIV infection were associated with unfavorable outcomes [31].

This study has two key limitations. First, in this secondary data analysis, some variables may not have been complete, which could have affected the analysis. Additionally, using a cross-sectional study design, we were able to establish a temporal relationship between the outcome and independent variables. Furthermore, because people with MDR-TB require a long time to complete treatment, approximately one-third of the patients were still in treatment when the data were analyzed, making it impossible to predict the exact treatment outcome for 2020 and 2021. The findings of this study on the factors that influence unfavorable outcomes among people infected with MDR-TB can help policymakers enhance MDR-TB control measures.

## Conclusion

This analysis revealed a high proportion of patients with unfavorable treatment outcomes among people with MDR-TB in Sierra Leone. Factors associated with unfavorable outcomes included previous TB treatment, HIV infection status, underweight status, age groups between 20 and 45 years and age groups between 46 and 65 years. Increasing patients’ awareness heightens treatment adherence, and HIV monitoring can reduce unfavorable outcomes in Sierra Leone and other similar settings. We recommend that to enhance mentoring and support for retreatment cases throughout the treatment journey, close follow-up and adherence support and counseling may be needed to address challenges that could lead to poor treatment outcomes.

## Data Availability

The datasets used and analyzed during the current study are available from the corresponding author upon reasonable request.
